# Compliance with the current recommendations for prescribing antibiotics for paediatric community-acquired pneumonia is improving: data from a prospective study in a French network

**DOI:** 10.1186/s12887-016-0661-3

**Published:** 2016-08-12

**Authors:** Elise Launay, Karine Levieux, Corinne Levy, François Dubos, Alain Martinot, Bénédicte Vrignaud, Flora Lepage, Robert Cohen, Emmanuel Grimprel, Matthieu Hanf, François Angoulvant, Christèle Gras-Le Guen, GPIP

**Affiliations:** 1 GPIP, Groupe de Pathologie Infectieuse Pédiatrique, Société Française de Pédiatrie, Paris, France; 2 Paediatric Department, CHU Nantes, Hôpital Mère-Enfant, 7 quai Moncousu, 44093 Nantes cedex 1, France; 3 Paediatric Emergency Department, CHU Nantes, Hôpital Mère-Enfant, Nantes, France; 4 ACTIV, Association Clinique et Thérapeutique Infantile du Val de Marne, Saint-Maur des Fossés, France; 5 Centre Hospitalier Intercommunal de Créteil, Centre de Recherche Clinique, Créteil, France; 6 Paediatric Emergency Unit & Infectious Diseases, Lille-2 Nord-de-France University & CHRU Lille, Lille, France; 7 Paediatric Department, AP-HP, Trousseau University Hospital, Paris, France; 8 Centre d’Investigation Clinique, CHU Nantes, Nantes, France; 9 INSERM CIE5, Clinical Epidemiology Unit, Université Paris Diderot, Sorbonne Paris Cité, Paris, France; 10 Paediatric Emergency Department, AP-HP, Hôpital Robert Debré, Paris, France

**Keywords:** Pneumonia, Antibiotic prescription, Antibiotic stewardship, Children

## Abstract

**Background:**

Lower respiratory tract infection is a common cause of consultation and antibiotic prescription in paediatric practice. The misuse of antibiotics is a major cause of the emergence of multidrug-resistant bacteria. The aim of this study was to evaluate the frequency, changes over time, and determinants of non-compliance with antibiotic prescription recommendations for children admitted in paediatric emergency department (PED) with community-acquired pneumonia (CAP).

**Methods:**

We conducted a prospective two-period study using data from the French pneumonia network that included all children with CAP, aged one month to 15 years old, admitted to one of the ten participating paediatric emergency departments. In the first period, data from children included in all ten centres were analysed. In the second period, we analysed children in three centers for which we collected additional data. Two experts assessed compliance with the current French recommendations. Independent determinants of non-compliance were evaluated using a logistic regression model. The frequency of non-compliance was compared between the two periods for the same centres in univariate analysis, after adjustment for confounding factors.

**Results:**

A total of 3034 children were included during the first period (from May 2009 to May 2011) and 293 in the second period (from January to July 2012). Median ages were 3.0 years [1.4–5] in the first period and 3.6 years in the second period. The main reasons for non-compliance were the improper use of broad-spectrum antibiotics or combinations of antibiotics. Factors that were independently associated with non-compliance with recommendations were younger age, presence of risk factors for pneumococcal infection, and hospitalization. We also observed significant differences in compliance between the treatment centres during the first period. The frequency of non-compliance significantly decreased from 48 to 18.8 % between 2009 and 2012. The association between period and non-compliance remained statistically significant after adjustment for confounding factors. Amoxicillin was prescribed as the sole therapy significantly more frequently in the second period (71 % vs. 54.2 %, *p* < 0.001).

**Conclusions:**

We observed a significant increase in the compliance with recommendations, with a reduction in the prescription of broad-spectrum antibiotics, efforts to improve antibiotic prescriptions must continue.

**Electronic supplementary material:**

The online version of this article (doi:10.1186/s12887-016-0661-3) contains supplementary material, which is available to authorized users.

## Background

In paediatrics, pneumonia frequently leads to medical consultations, antibiotic prescriptions, and hospitalization [1, 2]. In 2005, the French health authorities published guidelines for prescribing antibiotics, especially for community-acquired pneumonia (CAP) [3]. In these recommendations, amoxicillin is the first-line treatment recommended for suspected pneumococcal infections. Treatment with macrolides is recommended only in cases of subacute clinical presentation in older children. However, merely publishing recommendations is not a guarantee for compliance with them [4, 5]. Moreover, it has been shown that antibiotic resistance is closely linked to inadequate and improper antibiotic intake, highlighting the need for better ways to control antibiotic usage [6].

With growing concern about the widespread problems associated with antibiotic resistance, improved stewardship of paediatric antibiotics usage is being promoted in France through a national network (GPIP: Groupe de Pathologie Infectieuse Pédiatrique), in which most members have a particular interest in emergency care. In a study published 2012, Angoulvant et al. evaluated the impact of the antibiotic guidelines for acute respiratory-tract infection on antibiotic prescribing practice in a paediatric emergency department. They found a significant decrease of the antibiotic prescriptions from 32 to 21 % (*p* < 0.001, Cochran-Armitage test) [7]. Limiting the use of antibiotics is an effective way to reduce selection pressure, but when an antibiotic is indicated, in the case of pneumonia, for example, it is also important to prescribe the antibiotic appropriately with the optimal dosage and the narrowest spectrum that is likely to be effective. Indeed, guidelines for the management of upper respiratory tract infections, published in 2011, recommended a narrowing of the spectrum of antibiotics used. We hypothesized that these guidelines could have had an impact on the antibiotic prescription for CAP [8].

The aim of this study was to evaluate the extent and temporal course of non-compliance with current antibiotic prescription recommendations for paediatric CAP in paediatric emergency department (PED). We examined clinical practice in ten French PED, five years after the guidelines were published. The secondary objectives were to describe the cause of any medical non-compliance with these recommendations and to study the determinants of suboptimal prescribing practice by the doctor.

## Methods

### General methodology

We conducted a prospective two-period study using data from the French pneumonia network that included all children with CAP, aged one month to 15 years old, who presented at one of the ten participating PED [9]. CAP was assessed based on the association of a fever with consolidation on a chest X-ray [10]. All children admitted in PED, whether to be subsequently discharged home or hospitalized, were eligible for inclusion. The detailed methodology of this network is detailed elsewhere [9]. During the first period (from 1 May 2009 to 1 May 2011), we analysed the antibiotic prescriptions for children admitted to the ten PED. To evaluate the adequacy of the antibiotic prescriptions more accurately and add further detail to our initial results, we collected additional data during a second period (from 1 January 2012 to 1 July 2012) from three of the participating centres. These data concerned the patients’ characteristics and clinical features, as well as details about any antibiotic prescription. Exclusion criteria included the presence of empyema or the absence of data necessary to evaluate compliance with the prescribing guidelines.

The Robert Debré Hospital Ethics Committee approved the French pneumonia network, with informed consent waived.

### Collected data

Prospectively collected data available from the national network were demographic characteristics (age, sex); data about past medical history, particularly the presence of risk factors for pneumococcal disease (among them immune deficit, asplenia, sickle cell disease, chronic lung disease); clinical data (fever level, respiratory symptoms, presence of severity signs, such as respiratory distress or appearing ill); radiological data; microbiological data (blood culture); data concerning antibiotics prescribed in the PED (name and administration route); and data concerning outcome (hospitalization, apyrexia at 48 h follow-up and surviving status). Patients were followed for 48 h if seen as outpatients or followed until discharge if hospitalized. Deaths that occurred either in PED or during subsequent hospitalization were reported.

In period 2, some supplemental data were prospectively collected concerning clinical features helping to discriminate pneumococcal (sudden onset) from intracellular bacteria (subacute onset, myalgia or rash). More precise data about the antibiotic drug prescribed in the PED (dosage and duration), as well as data concerning the justification of the choice of antibiotic drug and its administration route (previous prescription of antibiotic for the same episode, allergy to antibiotic, vomiting, haemodynamic failure, or altered consciousness), were also collected. For feasibility reasons, these supplementary data were collected from only three chosen centres that comprised the largest proportion of study participants eligible for inclusion (representing 43 % of all inclusions in period 1).

### Assessment of compliance

Compliance with the prescription recommendations was assessed by 2 paediatric emergency physicians collectively (KL and FL). If there was any uncertainty, a third expert specializing in paediatric infectious diseases was asked to decide (CGL).

**Fig. 1:**
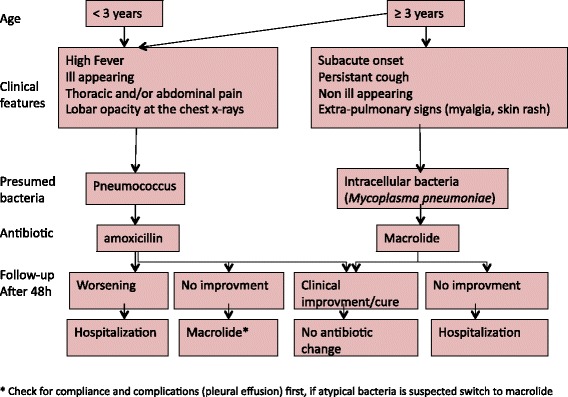
Algorithm of antibiotic choice recommended by the French health authorities in 2005

Compliance was evaluated according to the algorithm recommended by the health authorities (Fig. 1). Antibiotic prescription was considered as non-compliant according to these recommendations if: (i) a macrolide was prescribed as the first choice treatment in children younger than 3 years of age or in children with acute disease or septic signs, whatever their age; (ii) oral cephalosporin, cotrimoxazole, tetracycline, or azithromycin were prescribed as first line therapy; (iii) amoxicillin/clavulanate was prescribed in the absence of associated otitis or in children well-vaccinated against *Haemophilus infuenzae* serotype b; (iv) a combination of antibiotics (amoxicillin plus macrolide) was prescribed as a first line treatment in the absence of respiratory distress requiring intensive care; (v) an antibiotic was administered intravenously in the absence of known allergy to penicillin (which would justify the use of IV cephalosporin), and in the absence of severe respiratory distress, haemodynamic failure, altered consciousness, or vomiting; or (vi) if the dosage was insufficient compared to that recommended by health authorities (80–100 mg/kg/day for amoxicillin) [3].

To evaluate the non-compliance in period 1, the assessors used the following information: age, clinical features (respiratory distress, ill appearance, temperature), nature and administration route of the antibiotic drug prescribed. In period 2, the same information as in period 1 was used, as well information concerning (i) the onset of the disease (sudden—suggestive of *Pneumococcus*; or progressive—suggestive of atypical bacteria), (ii) the presence of vomiting, altered consciousness or haemodynamic failure justifying the use of IV antibiotic administration, (iii) the presence of allergy (justifying the use of a drug other than amoxicillin), (iv) the presence of extra-pulmonary symptoms (suggesting atypical bacteria), (v) the presence of associated otitis, (vi) the status for anti-*Haemophilus b* vaccine and (vii) the dosage of the antibiotic drug prescribed. Due to the lack of information concerning antibiotic drug dosage in period 1, this was not evaluated for compliance in period 1. In addition, as there was a lack of data concerning allergy in period 1, the IV administration of a third-generation cephalosporin was considered as non-compliant if administered in outpatients. Moreover, the use of amoxicillin–clavulanate was considered as non-compliant in period 1, as data on associated otitis or vaccination status for *Haemophilus* were lacking.

The development of the non-compliance between the two periods was assessed by comparing the frequency of non-compliant prescribing at the three centres included in both periods of the study.

### Statistical analysis

For the univariate analyses (comparing the compliant to non-compliant group or period 1 to period 2), we used either the chi-squared or Fisher’s exact test to compare the tabulated variables and either Student’s *t*-test or the Mann–Whitney *U*-test to compare the continuous variables. The potential determinants of non-compliance were age, signs of severe illness (defined as an ill appearance or respiratory distress), presence of risk factors for pneumococcal infection, hospitalization, centre, and qualification of the prescribers (for period 2 only). Independent factors associated with non-compliance with prescription recommendations were studied using multivariate analysis in the form of a logistic regression, selecting only those variables for which *p* < 0.1 in the univariate analysis. For period 1, we tested a hierarchical regression model that took into account the hierarchical structure of the data at a centre level (i.e., patients within a centre are not independent), which enabled us to construct a model for a ‘centre effect’. The hierarchical model could not be used in period 2 due to the small number of participating centres [11]. We compared the frequency of non-compliance for the three centres included in the two periods of the study with a chi-squared test for the univariate analysis. We adjusted the comparison of confounding factors (those previously identified as determinants of non-compliance) using logistic regression. As the duration and the seasons of collecting data were different (24 months in period 1 and 6 months in period 2), we tested the variability of non-compliance with seasons thanks to a chi-squared test (seasons were defined as winter from January to March, spring from April to June, summer from July to September and fall from October to December). We also included season as a potential confounder in the multivariate analysis. Quantitative variables were tested for linearity, transformed into polynomials, and categorized if they deviated. For analysis, we used Stata, v11 (StataCorp; College Station, TX, USA); a *p*-value of <0.05 was considered significant.

## Results

### Population characteristics

#### First period

During the first period, 3354 patients were seen in the PED network (including the 10 centres). We excluded 270 children with empyema and 50 missing data crucial to being evaluated for compliance with the recommendations. Amongst the remaining 3034 children, the median age was 3.0 years old and 32.2 % were hospitalized. Six children died (Table 1). Amoxicillin was prescribed in 58.1 % of the cases (Table 2).

**Table 1:** Demographics and clinical features of the children according to their compliance with recommendations and period of the study (numbers are percentages with denominators mentioned at the top of the column, except for those with superscript letters) with univariate and multivariate analyses for factors associated with prescription non-compliance (by period)

	Period 1									Period 2								
Variables	Total *N* = 3034	Compliant *N* = 1562	Non-compliant *N* = 1472	Univariate analysis			Multivariate analysis			Total *N* = 293	Compliant *N* = 237	Non-compliant *N* = 56	Univariate analysis			Multivariate analysis		
				OR	95 % CI	*p*	aOR	95 % CI	*p*				OR	95 % CI	*p*	aOR	95 % CI	*p*
Sex ratio	1.16	1.21	1.11			0.50				1	1.1	0.8			0.34			
Age																		
Median yrs [IQR]	3 [1.4–5]	3.4 [2–5.4]	2.3 [1–4.5]	0.2	0.1–0.3	<0.001*				3.6 [1.9–5.7]	3.4 [1.7–5.1]	4.5 [2.9–7.8]	1.1	1.01–1.2	0.008	1.2	1–1.3	0.007**
				10.7	5.7–20.1													
Age <1 year (%)	16.6	8.3	24.4	3.7	3–4.7	<0.001	3	2.4–3.9	<0.001	14	14.7	10.7	1	-	-			
Age >1 year (%)	83.4	91.7	74.6	1	-	-	1	-	-	86	85.3	89.3	1.4	0.6–3.6	0.44			
Severity signs																		
Respiratory distress (%)	28.5	20.5	36.5	2.3	1.9–2.7	<0.001	1	0.8–1.3	0.71	26.7	27.4	23.6	0.8	0.4–1.6	0.56			
Ill appearance (%)	31.3	22.1	39.9	2.5	2.1–2.9	<0.001	1.3	1.1–1.6	0.01	28.7	27.7	32.7	1.3	0.7–2.4	0.46			
Risk factors for pneumococcal infection^a^ (%)	5.7	3.5	8.1	2.4	1.8–3.5	<0.001	1.4	0.95–2.1	0.09	6.8	3.4	21.4	7.8	2.9–21.1	<0.001	4.1	1.3–12.7	0.02
Centres (%)																		
Centre 1	5.4	53.6	46.4	1	-	-	1	-	-									
Centre 2	12.4	60.6	39.4	0.8	0.5–1.1	0.13	0.9	0.5–1.3	0.37	34	85	15	1	-	-	1	-	-
Centre 3	20.5	48.8	51.2	1.2	0.9–1.7	0.27	1.2	0.8–1.8	0.48	32	87	13	2.4	1.2–4.9	0.01	1.3	0.6–3.1	0.53
Centre 4	13.1	36.6	63.4	2	1.4–2.9	<0.001	2	1.3–3	0.002									
Centre 5	4.8	22,1	77.9	4.1	2.4–6.9	<0.001	1.7	0.9–3.1	0.06									
Centre 6	12.3	65.5	34.5	0.6	0.4–0.9	0.009	0.6	0.4–0.9	0.02									
Centre 7	9.6	47.8	52.2	1.3	0.9–1.9	0.23	0.9	0.6–1.5	0.79	34	70	30	0.8	0.4–1.9	0.68	0.6	0.2–1.5	0.19
Centre 8	0.6	47.1	52.2	1.3	0.5–3.5	0.61	0.7	0.2–2.5	0.61									
Centre 9	17.8	63.5	36.5	0.7	0.5–0.9	0.02	0.7	0.5–1.1	0.17									
Centre 10	3.4	25.2	74.8	3.4	2–6	<0.001	1.9	1–3.6	0.05									
Season																		
Winter	25.3	25.3	25.2	1	-	-	1			78.5	76.4	87.5	1	-		1	-	-
Spring	17	18.4	15.6	0.7	0.6–0.9	0.01	0.7	0.5–0.9	0.012	21.5	23.6	12.5	0.5	0.2–1.1	0.07	0.5	0.2–1.4	0.18
Summer	15	15.4	14.7	0.8	0.7–1.6	0.15	0.8	0.6–1.1	0.25	-	-	-	-	-	-			
Fall	42.6	42.4	42.7	0.9	0.7–1.1	0.19	0.9	0.8–1.1	0.7	-	-	-	-	-	-			
Biology^b^ (%)																		
Leucocytes ≥10,000/mm^3^	74.2	76.2	73.5	0.9	0.6–1.1	0.26				68.9	72.9	61.1	0.6	0.2–1.4	0.21			
CRP ≥40 mg/L	53.9	55.6	53.8	0.9	0.7–1.1	0.18				55.2	55.8	54.1	0.9	0.4–2.1	0.86			
PCT ≥2 mg/mL	44.7	50	43.7	0.9	0.5–1.4	0.58				36.4	38.4	33.3	0.8	0.2–2.9	0.73			
Positive blood culture	3.6	3.2	3.8	1.2	0.5–2.8	0.71				4.9	2.9	7.7	3.2	0.3–39.6	0.33			
Prescriber (%)																		
Resident										53.4	44.4	55.5	1	-	-	1	-	-
Fellow										21.2	22.3	16.7	0.9	0.4–2.2	0.87	0.6	0.2–1.5	0.29
Senior										25.4	22.3	38.9	2.2	1.4–4.3	0.02	1.7	0.8–3.5	0.19
Hospitalization (%)	32.2	12.4	51.9	7.6	6.2–9.2	<0.001	5.7	4.6–7.2	<0.001	26.7	22	46.3	3.1	1.6–5.7	<0.001	4.1	2–8.4	<0.001
PICU	6.2	7.3	5.7							3.8	1.9	7.7						
Outcome (%)																		
Apyrexia at 48 h^c^	89.8	90	89.6	0.9	0.7–1.3	0.77				81.7	83.1	78.1	0.7	0.3–2	0.53			
Death	0.2	0.2	0.2	1	0.2–5.3	0.9				0.7	0.8	0						

Table 1 legend: *OR* odds ratio, *aOR* adjusted odds ratio, *95%CI* 95 % confidence interval, *IQR* interquartile range, *PICU* Paediatric Intensive Care Unit

*age was non-linear in period 1 and was transformed to a second degree polynomial as follows: x_1_ = ln(X) − 0.3 and *x*_2_ = X^0,5^ − 1.2 (where: X = age/1000) in the univariate analysis, age was entered as a dichotomous variable (around 1 year) in the multivariate analysis

**age was normally distributed in period 2 and was entered as a continuous variable in the multivariate analysis

^a^risk factors for pneumococcal infection were reported for 2992 children during period 1

^b^in period 1, white blood cell counts (WBC) were done for 1228 children, C-reactive protein (CRP) for 1394, procalcitonin (PCT) for 396, and blood cultures for 838 children; in period 2, WBC was done for 106 children, CRP for 114, and PCT for 44

^c^apyrexia occurring at 48 h was reported for 1640 children during period 1 and 115 children during period 2

**Table 2:** Description of the antibiotic prescriptions in the two periods of the study. n(%)

	Period 1 (10 centres)			Period 1 (3 centres)			Period 2 (3 centres)			p*
	Total	Compliant	Non-compliant	Total	Compliant	Non-compliant	Total	Compliant	Non-compliant	
	*N* = 3034	*N* = 1562	*N* = 1472	*N* = 1292	*N* = 672	*N* = 620	*N* = 293	*N* = 237	*N* = 56	
Mode										
None	126 (4.1)	0	126 (8.6)	9 (0.7)	0	9 (1.5)	1 (0.3)	0	1 (1.8)	0.5
Oral	2292 (75.5)	1558 (99.7)	734 (49.8)	1035 (80.6)	669 (99.7)	366 (59.0)	257 (87.7)	221 (92.9)	36 (27.3)	0.002
IV	616 (20.3)	4 (0.3)	612 (41.6)	248 (19.4)	3 (0.3)	245 (39.5)	35 (12)	16 (7.1)	19 (70.9)	0.003
Generic name										
Amoxicillin	1764 (58.1)	1526 (97.7)	238 (17.6)	700 (54.2)	655 (97.5)	45 (7.3)	208 (71)	203 (87.1)	5 (4.1)	<0.001
Amoxicillin + macrolide	98 (3.2)	3 (0.3)	95 (7.1)	49 (3.8)	1 (0.2)	48 (7.7)	13 (4.4)	0	13 (20.4)	0.6
3rd G cephalosporin	277 (9.1)	1 (0.1)	276 (19.5)	157 (12.2)	0	157 (25.3)	15 (5.1)	6 (2.6)	9 (18.3)	<0.001
Macrolide	93 (3.1)	(1.6)	68 (4.9)	46 (3.6)	10 (1.5)	36 (5.8)	16 (5.5)	11 (4.7)	5 (10.2)	0.13
Pristinamycin	6 (0.2)	5 (0.2)	1 (0.1)	5 (0.4)	4 (0.6)	1 (0.2)	2 (0.7)	0	2 (4.1)	0.5
C3G + Macrolide	17 (0.6)	0	17 (1.2)	13 (1)	0	13 (2.1)	0	0	0	0.09
Azithromycin	28 (1)	0	28 (2.1)	0	0	0	5 (1.7)	0	5 (10.2)	<0.001
Amoxicillin-Clavulanate	574 (18.9)	0	574 (40.1)	278 (21.5)	0	278 (44.8)	22 (7.5)	12 (5.2)	10 (16.3)	<0.001
Others	51 (1.7)	4 (0.3)	47 (7.4)	35 (2.71)	2 (0.3)	33 (5.3)	11 (3.8)	0	11 (16.4)	0.4

*p value of the comparison between the total antibiotic prescription in for the 3 centres participating to both periods (chi^2^)

#### Second period

During the second period, 300 patients were included from three participating centres (centres 2, 3, and 7). We excluded six children with empyema and one child for whom data about their antibiotic prescription(s) were missing. Among the 293 children that were included, the median age was 3.6 years old and 26.7 % were hospitalized. Two children died (Table 1). Amoxicillin was prescribed in 71.0 % of the cases (Table 2). The antibiotics were prescribed for 10 days for 73.4 % of the children, with a median prescribed duration of 10 days (ranging from 2 to 15 days).

### Compliance with recommendations

#### First period

Non-compliance with the current recommendations for antibiotic prescriptions occurred in 48.5 % of the cases included in the 10 centres. The most frequent reasons for non-compliance, in order of frequency, were as follows: the use of an antibiotic that was too broad-spectrum for the disease (amoxicillin-clavulanate or third generation cephalosporin) in 69.8 % of the non-compliant cases; unjustified intravenous (IV) administration in 37.2 %; unjustified concomitant administration of two or more antibiotics in 13.2 %; prescription of a macrolide despite amoxicillin being the preferred treatment in 4.8 %; and no antibiotics prescribed in 8.6 %. Among 126 children who did not receive antibiotics, 68 were described as ill-appearing or having respiratory distress signs and/or having risk factors for pneumococcal infection.

#### Second period

Non-compliance with the current recommendations for antibiotic prescriptions was seen in 18.8 % of the cases. The most frequent reasons for non-compliance, in order of frequency, were as follows: the use of unjustified concomitant administration of two or more antibiotics in 38.2 % of non-compliant cases; treatment that was too broad-spectrum for the disease (amoxicillin-clavulanate or third generation cephalosporin) in 36.4 %; prescription of a macrolide or pristinamycin despite amoxicillin being the preferred treatment in 9.1 %; the use of a macrolide not approved by the French medical agency for CAP indications in 9.1 %; unjustified IV administration in 3.6 % and insufficient dosage in 3.6 %;

### Determinants of non-compliance with recommendations

#### First period

In the univariate analysis, factors associated with non-compliance were as follows: an age of less than one year; signs that the disease was severe; the presence of risk factors for pneumococcal infection; centre of admission; and need for hospitalization. The antibiotic prescriptions were less often non-compliant during the spring season compared to winter season. In the multivariate analysis, independent factors significantly associated with non-compliance with prescriptions were as follows: an age of less than one year; being hospitalized; and admission into PED centre 4 or 10. Using a hierarchical regression model, the associations between non-compliance and age, and between non-compliance and hospitalization, were unchanged (the adjusted odds ratio [aOR] and 95 % confidence interval [CI 95 %] did not vary; see Additional file 1: Table S1). We did not find any difference in outcomes (apyrexia occurring at 48 h or death) between the compliant and non-compliant groups.

#### Second period

In the univariate analysis, factors associated with non-compliance were as follows: age (as a continuous variable); the presence of risk factors for pneumococcal infection; centre of admission; qualifications of the prescriber (seniority); and the need for hospitalization. Compliance did not significantly varied with season during the period 2 but there was a trend for less non-compliant prescription during spring as observed in period 1. In the multivariate analysis, independent factors significantly associated with non-compliance with prescriptions were: age (with a linear association); risk factors for pneumococcal infection; and hospitalization (Table 1). We did not find any differences between non-compliant and compliant groups and their association with the occurrence of apyrexia at 48 h.

### Comparison of the two periods

The three centres (2, 3 and 7 in Table 1) included in period 2 represented 1292 of the 3034 patients that were seen during period 1. If we consider only those three centres, non-compliance decreased from 48 % in period 1 to 18.8 % in period 2 (*p* < 0.001). Amoxicillin was prescribed as the sole therapy significantly more frequently in period 2 at these three centres (71 % vs. 54.2 %, *p* < 0.001) (Table 2). After adjustment for confounding factors, period 2 remained statistically associated with a lower risk of non-compliance (aOR = 0.2; 95 % CI: 0.1-0.3; *p* < 0.001) (Table 3). Considering only the 2 seasons (winter and spring) that were both studied during the 2 periods of the study, the period 2 remained independently associated with a lower risk of non-compliance with the same aOR (see Additional file 2: Table S2).

**Table 3:** Factors independently associated with non-compliance (results of the logistic regression). This analysis included only those children seen in the three centres participating in period 2 of the study

	Compliant	Non compliant	aOR	CI 95 %	*p*
	*N* = 909	*N* = 676			
	n (%)	n (%)			
Period					
First period	672 (52.0)	620 (48.0)	1	-	-
Second period	237 (80.9)	56 (18.5)	0.2	0.1–0.3	<0.001
Age					
< 1 year	97 (33.8)	190 (62.6)	1	-	-
> 1 year	812 (66.2)	486 (37.4)	0.4	0.3–0.5	<0.001
Risk factors for pneumococcal infection					
No	859 (59.2)	591 (40.8)	1	-	-
Yes	30 (21.3)	66 (68.7)	2.4	1.4–4.1	0.002
Respiratory distress					
No	687 (64.0)	386 (36.0)	1	-	-
Yes	207 (43.0)	275 (57.0)	0.8	0.6–1.1	0.62
Ill appearance					
No	679 (63.3)	394 (36.7)	1	-	-
Yes	208 (44.4)	260 (55.6)	1.3	0.9–1.7	0.11
Season					
Winter	353 (38.8)	233 (34.5)	1	-	-
Spring	173 (19)	106 (15.7)	0.8	0.6–1.1	0.21
Summer	105 (11.6)	95 (14)	1	0.7–1.5	0.97
Fall	278 (30.6)	242 (35.8)	0.9	0.6–1.2	0.44
Hospitalization					
No	783 (70.7)	324 (29.3)	1		
Yes	117 (25.2)	348 (74.8)	7	5.1–9.7	<0.001

## Discussion

In this prospective observational study, we found that non-compliance with recommendations for antibiotic prescriptions in paediatric CAP was frequent in paediatric emergency department, but decreased from the first to the second period (48 % vs. 18.8 %), with a greater use of amoxicillin as monotherapy at Period 2. Although compliance improved during this period, the main reasons for non-compliance were still the improper use of broad-spectrum antibiotics or combinations of antibiotics. Factors those were independently associated with the non-compliance with recommendations included younger age (less than 1 year old), presence of risk factors for pneumococcal infection, and hospitalization. Furthermore, we observed significant differences in compliance between the treatment centres during period 1.

The increase in amoxicillin use during the period of study could partly be the result of the Hawthorne effect, meaning that clinicians may have improved their compliance with the recommendations because they knew that they were being evaluated on this point. However, the GPIP network was not set up primarily to evaluate prescriptions, and the prescribers in each centre were not aware of the objectives of this ancillary study. The high proportion of non-compliance with prescriptions that occurred during period 1 could be partially due to a classification bias. Indeed, accurate information concerning the patients’ particularities (such as allergies, presence of comorbidities, etc.) was collected during the second period but not during the first period. Although these data could have justified the use of broad-spectrum antibiotics, this type of antibiotic was prescribed to a lesser extent during period 2. For example, the use of amoxicillin–clavulanate was considered as non-compliant in period 1, because information about associated otitis or vaccination status against *Haemophilus influenzae* was not available; in contrast, in period 2, such information was available and therefore could be taken into account, thus justifying the prescription of amoxicillin–clavulanate. However, amoxicillin–clavulanate was significantly less prescribed in period 2. This indicates that the prescriptions were more often judged as compliant, due to an actual improvement in adhering to the guidelines, rather than a change in the way compliance was evaluated during the two periods of the study. The increase in amoxicillin use observed in our study between periods 1 and 2 is consistent with the findings reported in a study by Smith et al., which showed that, after the implementation of guidelines recommending the use of amoxicillin, prescription rates of amoxicillin for CAP increased from 2 to 44 % [12].

We observed that younger ages were associated with a higher risk of non-compliance in period 1, whereas, by contrast, the aOR for non-compliance was 1.2 for each year of age in period 2. This could be attributed to the increased likelihood that during the first period, young children were more likely to receive amoxicillin-clavulanate than in the second period. The use of amoxicillin-clavulanate could be explained by the fear of invasive *Haemophilus influenzae* type b. We also observed that children with risk factors for invasive pneumococcal infection were more likely to be subject to non-compliant prescriptions, particularly during period 2. We hypothesize that doctors might fear, in an irrational manner, encountering resistant pneumococci in these children. Indeed, since the more widespread use of the pneumococcal vaccine, resistance to penicillin has decreased, as described in a previous Palestinian–Israeli study [13]. This decrease in the resistance to penicillin was also observed in France, with 82 % of the 1858 strains analysed by the national reference centre being susceptible to amoxicillin in 2009 vs. 92 % of the 957 analysed strains in 2012 (*p* < 0.001) [14, 15]. The use of broad spectrum antibiotics was therefore needed less often. It has also been shown that antibiotic guidelines for upper respiratory tract infections, published on November 2011 in France, had a major impact on antibiotic prescriptions, resulting in increased amoxicillin use [14]. These recommendations could have also impacted the antibiotic prescriptions for pneumonia in our study by sensitizing prescribers to the need to use narrow-spectrum antibiotics in order to limit the risk of resistant bacteria.

The absence of antibiotic prescription was considered as non-compliant. It could be argued that children who did not receive antibiotics were suspected of having a viral infection, which therefore obviated the need for antibiotics. French health authorities recommended, in 2005, to perform a chest X-ray in cases where CAP is suspected and to treat all cases of CAP with antibiotics, as the differentiation between viral and bacterial infections is not easy to distinguish [3]. The latest guideline from the British Thoracic Society also recommends for all children with pneumonia to be treated with antibiotics; however, the Society also stated that fully vaccinated children of less than 2 years old of age with mild lower respiratory symptoms are not likely to have bacterial pneumonia (but rather viral pneumonia) and therefore require no chest X-rays or antibiotics [16]. The American recommendations from the Pediatric Infectious Diseases Society and the Infectious Diseases Society of America also stated that ‘Antimicrobial therapy is not routinely required for preschool-aged children with CAP, because viral pathogens are responsible for the great majority of clinical disease’ (strong recommendation; high-quality evidence) [17]. Therefore, challenging the diagnosis of bacterial pneumonia in fully vaccinated children younger than 2 years old with mild symptoms is another way to reduce antibiotic use.

Assessors did not take into account the prescribed duration of antibiotics in their evaluation of compliance, as the information was not available during period 1. The French recommendations are to treat for 10 days if a pneumococcal infection is suspected, whereas the American guidelines recommend the shorter effective treatment. A systematic review, published in 2008, found evidence that a short course of 3 days was as effective as a longer course of 5 days of antibiotics for children younger than 5 years old with non-severe CAP in developed countries. However, these results could not be generalized, as the inclusion criteria were not restricted to bacterial pneumonia [18]. Therefore, the shorter effective treatment still remains to be defined in developed countries.

This study has several limitations. Firstly, the evaluation of compliance was not done independently by several experts; however, the existence of precise guidelines made the evaluation less subjective. Secondly, some data were missing, but this was only an issue for 50 children in period 1 (1.6 %) and one in period 2 (0.3 %). Thirdly, the 10 centres from period 1 (particularly those with the highest aOR) were not all included in period 2, risking undervaluation for non-compliance during period 2. Nevertheless, the mean non-compliance from period 1 was very similar in these three centres to the mean of all ten: 48.0 % compared with 48.5 %. Finally the 2 periods of the study differed in their length with 4 seasons covered in period 1 and only 2 seasons in period 2. This could have limited the relevance of the comparison between the 2 periods. To avoid this potential risk of bias we adjusted the comparison between the two periods on the seasons and we also made a comparison restricted to the 2 seasons that were both studied in the 2 periods. In the 2 cases, we found a significant decrease of non-compliance in period 2 with an aOR at 0.2 (95 % CI 0.1–0.3).

## Conclusion

A reduction in the prescription of broad-spectrum antibiotics is feasible by reducing the use of broad-spectrum drugs that are no longer justified in the treatment of ‘typical’ CAP, since the incidence of penicillin-resistant *Pneumococcus* is decreasing [13], by avoiding overtreatment of viral pneumonia with antibiotics [17, 18] and by reducing the treatment duration to a shorter effective course [19].

## Abbreviations

aOR, adjusted odds ratio; GPIP, Groupe de Pathologie Infectieuse Pédiatrique; CAP, community-acquired pneumonia; PED, paediatric emergency department

## Additional files

Additional file 1: Table S1. Multivariate analysis testing the independent association between the variables and noncompliance with prescriptions using hierarchical regression model (center effect) during the first period. (DOCX 22 kb) Additional file 2: Table S2. Factors independently associated with non-compliance (results of the logistic regression). This analysis included only those children seen in the three centres participating in period 2 of the study during winter or spring. (DOCX 73 kb)
