# Supplementary material for: Compliance with the current recommendations for prescribing antibiotics for paediatric community-acquired pneumonia is improving: data from a prospective study in a French network
Source: BMC Pediatr. 2016 Aug 12;16:126. doi: 10.1186/s12887-016-0661-3 (PMC4983061; doi:10.1186/s12887-016-0661-3)
Supplement: Additional file 1: Table S1. — Multivariate analysis testing the independent association between the variables and noncompliance with prescriptions using hierarchical regression model (center effect) during the first period. (DOCX 22 kb) [file 12887_2016_661_MOESM1_ESM.docx]

**Additional Table 1**

Multivariate analysis testing the independent association between the variables and noncompliance with prescriptions using hierarchical regression model (center effect) during the first period

|  | aOR | 95% CI | p |
| --- | --- | --- | --- |
| **Age** |  |  |  |
| Age <1 yr | 3 | 2.4–3.9 | <0.001 |
| Age >1 yr | 1 | - | - |
| **Respiratory distress** |  |  |  |
| No | 1 | - | - |
| Yes | 1 | 0.8–1.3 | 0.75 |
| **Ill appearance** |  |  |  |
| No | 1 | - | - |
| Yes | 1.31 | 1.1–1.6 | 0.01 |
| **Risk factors for pneumococcal infection** |  |  |  |
| No | 1 | - | - |
| Yes | 1.4 | 0.96–2.1 | 0.08 |
| **Hospitalization** |  |  |  |
| No | 1 | - | - |
| Yes | 5.8 | 4.6–7.2 | <0.001 |
| **Seasons** |  |  |  |
| Winter | 1 | - | - |
| Spring |  |  |  |
| Summer |  |  |  |
| Fall |  |  |  |

aOR: adjusted odds ratio, 95%CI: 95% confidence interval
