# Supplementary material for: Compliance with the current recommendations for prescribing antibiotics for paediatric community-acquired pneumonia is improving: data from a prospective study in a French network
Source: BMC Pediatr. 2016 Aug 12;16:126. doi: 10.1186/s12887-016-0661-3 (PMC4983061; doi:10.1186/s12887-016-0661-3)
Supplement: Additional file 2: Table S2. — Factors independently associated with non-compliance (results of the logistic regression). This analysis included only those children seen in the three centres participating in period 2 of the study during winter or spring. (DOCX 73 kb) [file 12887_2016_661_MOESM2_ESM.docx]

**Additional Table 2**

Factors independently associated with non-compliance (results of the logistic regression). This analysis included only those children seen in the three centres participating in period 2 of the study during winter or spring.

|  | Compliant  N= 526  n (%) | Non compliant  N= 339  n (%) | aOR | CI 95% | p |
| --- | --- | --- | --- | --- | --- |
| **Period** |  |  |  |  |  |
| First period |  |  | 1 | - | - |
| Second period |  |  | 0.2 | 0.1-0.3 | <0.001 |
| **Age** |  |  |  |  |  |
| < 1 year |  |  | 0.4 | 0.2-0.6 | <0.001 |
| > 1 year |  |  | 1 | - | - |
| **Risk factors for pneumococcal infection** |  |  |  |  |  |
| No | 507 (97.3) | 300 (89.8) | 1 | - | - |
| Yes | 14 (2.7) | 34 (10.2) | 3.8 | 1.7-3.1 | 0.001 |
| **Respiratory distress** |  |  |  |  |  |
| No | 397 (76.6) | 195 (59.1) | 1 | - | - |
| Yes | 121 (23.4) | 135 (40.9) | 0.8 | 0.5-1.2 | 0.20 |
| **Ill appearance** |  |  |  |  |  |
| No | 386 (75.1) | 193 (59) | 1 | - | - |
| Yes | 128 (24.9) | 134 (41) | 1.2 | 0.8-1.7 | 0.41 |
| **Season** |  |  |  |  |  |
| Winter | 353 (67.1) | 233 (68.7) | 1 | - | - |
| Spring | 173 (32.9) | 106 (31.3) | 0.8 | 0.5-1.1 | 0.20 |
| **Hospitalization** |  |  |  |  |  |
| No | 446 (85.4) | 152 (45.1) | 1 | - | - |
| Yes | 76 (14.6) | 185 (54.9) | 7.6 | 4.9-11.8 | <0.001 |
